# Biofluid‐based biomarkers for tau pathophysiology in Alzheimer's disease

**DOI:** 10.1002/alz70856_099493

**Published:** 2025-12-24

**Authors:** Henrik Zetterberg, Kaj Blennow

**Affiliations:** ^1^ University of Gothenburg, Mölndal, Gothenburg, Sweden; ^2^ Department of Psychiatry and Neurochemistry, Institute of Neuroscience and Physiology, The Sahlgrenska Academy, University of Gothenburg, Mölndal, Sweden

## Abstract

**Background:**

Different forms of tau proteins can be measured in biofluids such as cerebrospinal fluid (CSF), plasma, and saliva. Research during the past two decades have established how the biomarker works.

**Method:**

Ultrasensitive immunoassays and mass spectrometry methods.

**Result:**

Tau proteins in biofluids can be N‐terminal and phosphorylated. These forms of tau mainly reflect and amyloid‐induced increased secretion of tau from neurons and are hence quite specific biomarkers for Alzheimer's disease (AD). They are not increased in primary tauopathies like progressive supranuclear palsy or corticobasal degeneration. They predict AD‐type tangle pathology but are not direct markers of this pathology. They normalize in response to anti‐amyloid treatment, but not completely. More C‐terminal forms of tau can also be measured in biofluids. Some of these fragments seem to more strongly correlate with tangle pathology, as determined by tau positron emission tomography. AD correlations are mainly seen in CSF and plasma, whilst other biofluids like saliva do not appear to be useful sample types.

**Conclusion:**

The presentation will summarize the most important findings explaining what biofluid‐based tau protein concentrations reflect and how these markers can be used alongside tau‐PET to improve the diagnostics, staging and therapy‐monitoring in AD.

